# Cost-effectiveness of first-line immunotherapy combinations with or without chemotherapy for advanced non-small cell lung cancer: a modelling approach

**DOI:** 10.1186/s12885-024-12287-6

**Published:** 2024-07-22

**Authors:** Ash Bullement, Mark Edmondson-Jones, Nicholas Latimer

**Affiliations:** 1 Delta Hat, Nottingham, UK; 2 SCHARR, University of Sheffield, Sheffield, UK; 3 Population Health Sciences, University of Leicester, Leicester, UK

**Keywords:** Cost-effectiveness, Non-small cell lung cancer, Partitioned survival model, Hazard ratio, Proportional hazards, Network meta-analysis, Survival analysis

A recent modelling study by Hui et al. considered a cost-effectiveness analysis of 11 different treatment options for patients with advanced non-small cell lung cancer (NSCLC) [1]. The task set out by the authors is no small feat, given the need to synthesise evidence from many sources in order to produce relevant comparisons. Nevertheless, such types of study warrant carefully considered analyses of the evidence available to inform suitable estimates of relative effects, in order to appropriately inform cost-effectiveness results. On review we have several concerns with the methodological approach taken to produce the cost-effectiveness results, described below.

First, the authors consider findings from a published network meta-analysis (NMA) of first-line immunotherapy combinations for advanced NSCLC by Liu et al., (2021) [2]. In neither the NMA study, nor this cost-effectiveness study, is any evidence of testing for proportional hazards (PH) provided. Assessment of the PH assumption is critical to determining if the use of hazard ratios (HRs) within an NMA framework is appropriate to derive suitable relative efficacy estimates, particularly if these HRs are intended to be used to inform lifetime extrapolations of survival outcomes. This would normally take place at the feasibility stage of the NMA but is not described by Liu and colleagues. This modelling study ultimately applies the PH assumption throughout the network – a large assumption given the survival profile of immunotherapies, and the inclusion of different types of NSCLC within the network (e.g., squamous and non-squamous sub-types).

The authors selected the atezolizumab + chemotherapy arm of the IMpower130 trial (NCT02367781) as the baseline treatment, from which estimates of treatment effect were applied from the NMA. This study was selected *“due to its large sample, long follow-up time, and stable result”* [1]. While in principle this rationale is sound, other features of IMpower130 lead us to question the authors’ decision to base their analyses on a PH assumption. The Kaplan-Meier (KM) estimates of overall survival (OS) for the treatment groups being compared in IMpower130 cross multiple times over the first ∼ 2 months of follow-up, and this is not just the case for IMpower130 [3]. For example, in the CheckMate-227 trial (NCT02477826), there is a clear crossing of the KM estimates of OS for the treatment groups being compared at around 6 months [4], and a similar finding is observed in the MYSTIC trial (NCT02453282), where KM estimates for both OS and progression-free survival (PFS) cross at around 6 months [5].

Crossing KM estimates may indicate that the ratio of event hazards between the treatments being compared are not proportional, rendering the HR a questionable means of assessing the difference in survival between treatment groups. However, the extent to which the KM estimates cross may not be sufficient grounds to reject the PH assumption, and so further inspection of the data informing the NMA is necessary. There are a number of tools available to aid with assessing the suitability of the PH assumption beyond inspection of the KM estimates, such as log-cumulative hazard plots and Schoenfeld residuals [6]. The issue of violating the PH assumption is exacerbated in the context of this modelling study, since it is assumed to hold for the comparison of 11 different treatment regimens. If even one of the studies that forms a connection within the network provides an unsuitable estimate of the relative effect between treatment regimens, the plausibility of the full NMA is questionable. The structure of the network used to inform this analysis relies heavily on the PH assumption holding between treatment groups that received immunotherapy (either alone, or in combination with chemotherapy) versus chemotherapy alone. Several previous studies have described the issue of PH not holding when comparing treatment regimens with different mechanistic properties, and it is generally accepted that immunotherapies can have delayed but durable treatment effects, resulting in hazard functions and survival curves that have different shapes to those associated with chemotherapies [7–12].

Secondly, setting aside concerns regarding the PH assumption underpinning the NMA, the authors use the outputs from the NMA to inform the estimates of OS and PFS for the other ten regimens considered within their analysis. The authors explain that *“Based on the* [PH] *assumption, the log-logistic distribution was also used to fit and extrapolate the PFS and OS curves for the other ten treatment regimens.”* Here, it is important to highlight that the selection of the log-logistic model is directly at odds with the assumption of PH. The log-logistic model is an accelerated failure time (AFT) model, and so treatment effects are assumed to impact the *time* ratio, not the *hazard* ratio. It is not possible to apply HRs to a log-logistic model (i.e., an AFT model), as described in the authors’ Eq. 5, and have the resultant model still reflect a log-logistic form, as implied by the authors: *“On the basis of the derivation above, the common shape of the OS curve and the common shape of the PFS curve for the 11 treatment regimens were 1.3 and 1.7 respectively”* [1].

Finally, outside the context of the estimation of relative effects, the authors make further assumptions regarding the estimation of costs and quality-adjusted life years. For example, all immunotherapies were assumed to be given to all patients with progression-free disease up until 2 years. Some patients with progression-free disease may stop treatment due to unacceptable toxicity, though this is not discussed, and some may continue beyond 2 years (either in the clinical trial or in ‘real-world’ practice). Furthermore, a utility value of 0.321 was assumed to apply for the progressed disease health state. This value is not representative of the estimates used to inform other, recent cost-effectiveness analyses in NSCLC (see, for example, Beca et al., [2021] [13] and Jiang & Wang [2022] [14]), or indeed many other cost-effectiveness studies of cancer populations (e.g., Morimoto et al. [2022] [15] in unresectable metastatic pancreatic cancer which included a post-progression utility value of 0.75) though no explanation is given for this.

Overall, while all modelling studies are subject to limitations, this study appears to have methodological flaws which mean that the conclusions are prone to substantial error, expected bias, and are unlikely to be reliable. We also do not believe the authors have adequately discussed some critical issues as a part of their analysis. With regards to the NMA approach, other techniques are available to consider indirect comparisons in the presence of non-PH – for example, a non-PH NMA by Herbst et al. of cancer immunotherapies versus chemotherapy for first-line treatment of patients with NSCLC and high programmed death-ligand 1 expression made use of non-PH fractional polynomial models within a Bayesian framework [16]. For the other aspects of the analysis that we highlight (related to costs and utilities), it may have been helpful to further explore the available discontinuation data from each study to determine if a 2-year stopping rule is appropriate for all cases, and alternative utility values from the literature may have been worthy of consideration – for example, Blom et al. undertook a systematic review and meta-analysis of utility values for lung cancer [17].

Regardless of the ultimate approach taken, the presence of other methodologies warrant discussion with respect to the strengths and limitations of the analyses undertaken. While it is encouraging to see research aiming to produce cost-effectiveness evidence for decision making, it is concerning that the authors reach strong conclusions regarding which regimens appear to be the most cost effective, and that the analysis serves as *“evidence for pharmaceutical enterprises to properly and deeply consider the pricing strategy based on effectiveness and safety in the real-world condition”* [1]. Making such claims of the basis of an analysis which lacks robustness should be caveated accordingly.

## Data Availability

Not applicable.

## Abbreviations

AFT: Accelerated failure time
KM: Kaplan-Meier
NMA: Network meta-analysis
NSCLC: Non-small cell lung cancer
OS: Overall survival
PFS: Progression-free survival
PH: Proportional hazards

## References

[1] Hui W, Song R, Tao H, Gao Z, Zhu M, Zhang M, et al. Cost-effectiveness of first-line immunotherapy combinations with or without chemotherapy for advanced non-small cell lung cancer: a modelling approach. BMC Cancer. 2023;23:442. 37189081 10.1186/s12885-023-10938-8 PMC10186643

[2] Liu L, Bai H, Wang C, Seery S, Wang Z, Duan J, et al. Efficacy and safety of First-Line Immunotherapy combinations for Advanced NSCLC: a systematic review and network Meta-analysis. J Thorac Oncol. 2021;16:1099–117. 33839365 10.1016/j.jtho.2021.03.016

[3] West H, McCleod M, Hussein M, Morabito A, Rittmeyer A, Conter HJ, et al. Atezolizumab in combination with carboplatin plus nab-paclitaxel chemotherapy compared with chemotherapy alone as first-line treatment for metastatic non-squamous non-small-cell lung cancer (IMpower130): a multicentre, randomised, open-label, phase 3 trial. Lancet Oncol. 2019;20:924–37. 31122901 10.1016/S1470-2045(19)30167-6

[4] Brahmer JR, Lee J-S, Ciuleanu T-E, Bernabe Caro R, Nishio M, Urban L, et al. Five-year survival outcomes with Nivolumab Plus Ipilimumab Versus Chemotherapy as First-Line Treatment for Metastatic non–small-cell Lung Cancer in CheckMate 227. J Clin Oncol. 2023;41:1200–12. 36223558 10.1200/JCO.22.01503 PMC9937094

[5] Rizvi NA, Cho BC, Reinmuth N, Lee KH, Luft A, Ahn M-J, et al. Durvalumab with or without Tremelimumab vs Standard Chemotherapy in First-line treatment of Metastatic non–small cell Lung Cancer: the MYSTIC Phase 3 Randomized Clinical Trial. JAMA Oncol. 2020;6:661–74. 32271377 10.1001/jamaoncol.2020.0237 PMC7146551

[6] Schoenfeld D. Partial residuals for the proportional hazards regression model. Biometrika. 1982;69:239–41. 10.1093/biomet/69.1.239

[7] Chen T-T. Statistical issues and challenges in immuno-oncology. J Immunother Cancer. 2013;1:18. 24829754 10.1186/2051-1426-1-18 PMC4019889

[8] Royston P, Parmar MK. An approach to trial design and analysis in the era of non-proportional hazards of the treatment effect. Trials. 2014;15:314. 25098243 10.1186/1745-6215-15-314 PMC4133607

[9] Dehbi H-M, Royston P, Hackshaw A. Life expectancy difference and life expectancy ratio: two measures of treatment effects in randomised trials with non-proportional hazards. BMJ. 2017;357:j2250. 28546261 10.1136/bmj.j2250 PMC5444092

[10] Rahman R, Fell G, Ventz S, Arfé A, Vanderbeek AM, Trippa L, et al. Deviation from the proportional hazards Assumption in Randomized Phase 3 clinical trials in Oncology: Prevalence, Associated Factors, and implications. Clin Cancer Res. 2019;25:6339–45. 31345838 10.1158/1078-0432.CCR-18-3999

[11] Ding X, Wu J. Designing cancer immunotherapy trials with delayed treatment effect using maximin efficiency robust statistics. Pharm Stat. 2020;19:424–35. 32090453 10.1002/pst.2003 PMC9423373

[12] Quinn C, Garrison LP, Pownell AK, Atkins MB, de Pouvourville G, Harrington K, et al. Current challenges for assessing the long-term clinical benefit of cancer immunotherapy: a multi-stakeholder perspective. J Immunother Cancer. 2020;8:e000648. 32661115 10.1136/jitc-2020-000648 PMC7359062

[13] Beca JM, Walsh S, Raza K, Hubay S, Robinson A, Mow E, et al. Cost-effectiveness analysis of first-line treatment with crizotinib in ROS1-rearranged advanced non-small cell lung cancer (NSCLC) in Canada. BMC Cancer. 2021;21:1162. 34715804 10.1186/s12885-021-08746-z PMC8556902

[14] Jiang Y, Wang X. Cost-effectiveness analysis of pembrolizumab plus standard chemotherapy versus chemotherapy alone for first-line treatment of metastatic non-squamous non–small-cell lung cancer in China. Eur J Hosp Pharm. 2022;29:139–44. 32737070 10.1136/ejhpharm-2020-002208 PMC9047884

[15] Morimoto K, Moriwaki K, Kaneyasu T, Nakayama H, Shimozuma K. Cost-effectiveness of Nab-Paclitaxel and Gemcitabine Versus Gemcitabine Monotherapy for patients with unresectable metastatic pancreatic Cancer in Japan. Value Health Reg Issues. 2022;28:54–60. 34800832 10.1016/j.vhri.2021.04.003

[16] Herbst R, Jassem J, Abogunrin S, James D, McCool R, Belleli R et al. A Network Meta-Analysis of Cancer immunotherapies Versus Chemotherapy for First-Line treatment of patients with Non-small Cell Lung Cancer and high programmed death-ligand 1 expression. Front Oncol. 2021;11. 10.3389/fonc.2021.676732 PMC8300186 34307144

[17] Blom EF, ten Haaf K, de Koning HJ. Systematic review and Meta-analysis of community- and choice-based Health State Utility values for Lung Cancer. PharmacoEconomics. 2020;38:1187–200. 32754857 10.1007/s40273-020-00947-x PMC7547043

